# Supplementary figures and images for: An Integrative Systems Biology and Experimental Approach Identifies Convergence of Epithelial Plasticity, Metabolism, and Autophagy to Promote Chemoresistance
Source: J Clin Med. 2019 Feb 7;8(2):205. doi: 10.3390/jcm8020205 (PMC6406733; doi:10.3390/jcm8020205)

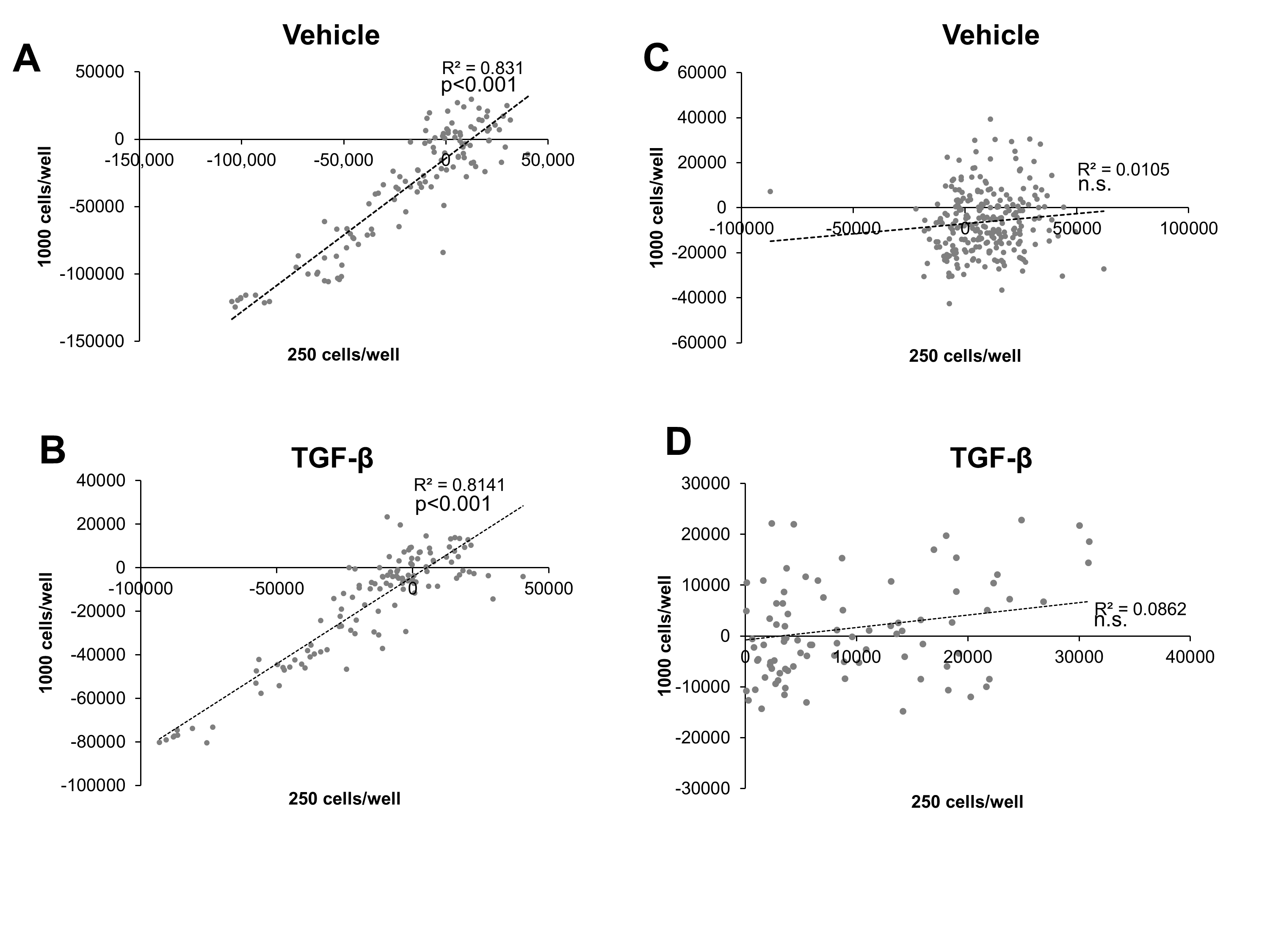

Supplement: Supplementary file 1 [file jcm-08-00205-s001.zip › Supplementary Figure 1.tif]
